# Several flat tendon graft types are viable options for flat superficial medial collateral ligament reconstructions—A biomechanical analysis

**DOI:** 10.1002/ksa.12705

**Published:** 2025-05-19

**Authors:** Thorben Briese, Christian Peez, Philipp Runde, Matthias Klimek, Adrian Deichsel, Michael J. Raschke, Christoph Kittl, Elmar Herbst

**Affiliations:** ^1^ Department of Trauma, Hand and Reconstructive Surgery University Hospital Muenster Muenster Germany

**Keywords:** flat MCL, flat tendon, MCL, MCL graft, medial collateral ligament

## Abstract

**Purpose:**

Flat superficial medial collateral ligament (sMCL) reconstruction helps restore knee kinematics in medial instability, but recommendations on grafts that best mimic the sMCL's biomechanical properties are missing. This study aimed to compare the biomechanical properties of flat grafts to the native sMCL, hypothesizing that (1) flat grafts exhibit unique biomechanical properties and (2) graft configuration affects their biomechanical properties.

**Study Design:**

Controlled laboratory study.

**Methods:**

The sMCL, semitendinosus, gracilis, quadriceps tendons and iliotibial band (ITB) were harvested from 20 fresh‐frozen human cadaveric knees. Flat grafts were prepared, providing single‐ and double‐strand grafts. The following groups (*n* = 10 each) were defined: (1) native sMCL, (2) single‐strand semitendinosus tendon (SemiT single), (3) double‐strand semitendinosus tendon (SemiT double), (4) single‐strand gracilis tendon (Gracilis single), (5) double‐strand gracilis tendon (Gracilis double), (6) single‐strand ITB (ITB single), (7) double‐strand ITB (ITB double) and (8) superficial layer of the quadriceps tendon (Quad). Using a universal uniaxial testing machine, the grafts were preconditioned (10 cycles, 10–50 N) and subsequently loaded to failure (LTF) (20 mm/min). Biomechanical properties of the grafts were compared using a one‐way analysis of variance with post hoc correction (*p* < 0.05).

**Results:**

Double‐strand configuration of the hamstring tendons and the ITB resulted in a significant increase in stiffness, LTF and yield load (*p* < 0.05) and a significant decrease in tensile stress and ultimate strain (*p* < 0.05) compared to single‐strand grafts. Single‐strand SemiT and Quad, as well as double‐strand Gracilis and ITB grafts, demonstrated comparable biomechanical properties to the native sMCL (n.s.). In contrast, double‐strand SemiT tendon grafts exhibited significantly greater stiffness, LTF and yield load compared to the native sMCL (*p* < 0.01). Conversely, single‐strand graft configuration of Gracilis and ITB resulted in significantly lower stiffness, LTF and yield load (*p* < 0.01) and higher tensile stress and ultimate strain (*p* < 0.001) compared to the native sMCL.

**Conclusion:**

The graft type and configuration of the grafts significantly affects the biomechanical properties of flat sMCL grafts. Single‐strand SemiT and Quad, and double‐strand Gracilis and ITB grafts, mimic the biomechanical properties of the native sMCL, representing viable options for flat sMCL reconstructions. In contrast, double‐strand SemiT grafts exceeded these properties, while single‐strand Gracilis and ITB grafts demonstrate decreased biomechanical properties, possibly resulting in increased risk of medial overconstraint or residual laxity.

**Level of Evidence:**

There is no level of evidence as this study was an experimental laboratory study.

**Abbreviations**
ACL: anterior cruciate ligament
ANOVA: analysis of variance
ITB: iliotibial band
LTF: load to failure
MCL: medial collateral ligament
n.s.: not significant
Quad: quadriceps tendon
SD: standard deviation
SemiT: semitendinosus tendon
sMCL: superficial medial collateral ligament

## INTRODUCTION

Medial collateral ligament (MCL) injuries account for 7.9% of all knee injuries [19], with high‐grade MCL injuries often occurring concomitantly with anterior cruciate ligament (ACL) tears [13, 19, 32]. Such combined ACL and medial injuries can lead to persistent anteromedial instability [15, 16], which negatively affects patient‐reported outcomes and increases the risk of treatment failure after ACL reconstruction [2, 3, 4, 30]. With an improved understanding of the reciprocal function of the different fibre regions of the broad native sMCL [17, 33], biomechanical studies have demonstrated the advantages of flat medial reconstruction techniques to improve restoration of native knee kinematics in patients with persistent medial instability [8].

Despite advances in surgical techniques, there is a lack of evidence regarding the choice of graft that best mimics the anatomy and biomechanical characteristics of the native MCL complex [1, 23]. Recent studies on flat sMCL reconstructions used semitendinosus, gracilis or peroneus longus grafts [1, 8, 24], and previous studies have already examined the primary stability of natively flat grafts [5, 12, 21, 29]. However, research on surgically flattened round tendons was only performed on porcine models [9], and studies analyzing stability have not yet addressed the biomechanical properties relative to the sMCL and further flat graft options. Therefore, there is currently no clear evidence‐based recommendation for graft selection in flat sMCL reconstructions.

The purposes of this biomechanical study were (1) to characterize the biomechanical properties of common flat graft choices for flat sMCL reconstructions including flattened round human tendons, (2) to identify flat tendon grafts best mimicking the biomechanical behaviour of the native sMCL. It was hypothesized that (1) common flat tendon grafts provide unique biomechanical properties compared to the native sMCL and (2) graft configuration affects their biomechanical properties.

## MATERIALS AND METHODS

Twenty fresh‐frozen human cadaveric knees (mean age 79 ± 9 years; 10 females, 10 males; all Caucasian) with no history of previous ligamentous or bony injury were obtained from an international tissue bank (MedCure). The knee specimens were dissected and biomechanically tested with permission from the Institutional Review Board of the University of Muenster (Reference No. 2020‐181‐f‐S).

### Study groups and specimen preparation

**Figure 1:**
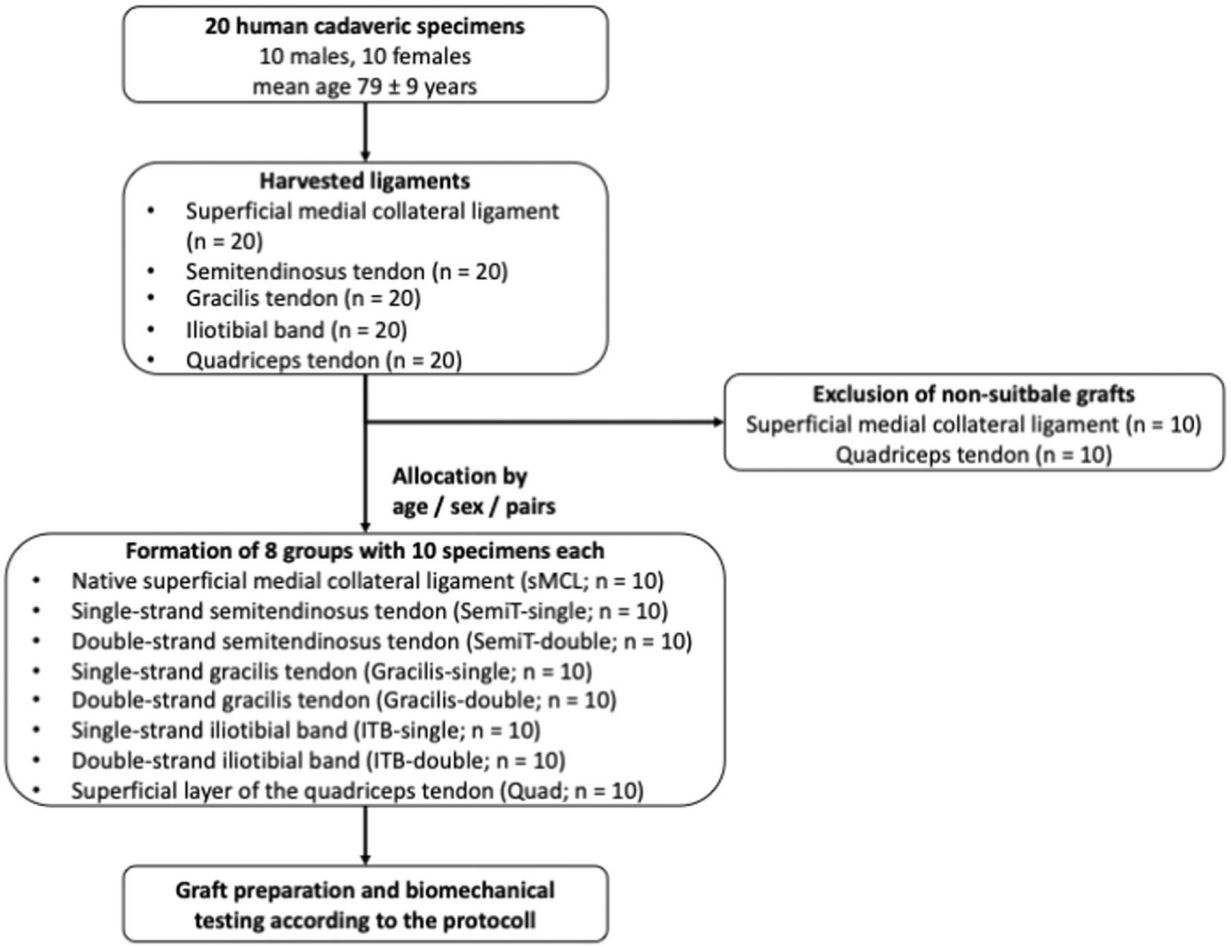
Flow chart of allocation and randomization of the grafts.

The sMCL and the tendon grafts were assigned to eight groups consisting of ten specimens each. The grafts were matched for demographics, so that there was a homogeneous distribution of age and sex among the eight groups between the testing conditions (Figure 1). Based on this, the following eight groups were formed:

(1) native superficial medial collateral ligament (sMCL)
(2) single‐strand semitendinosus tendon (SemiT single)
(3) double‐strand semitendinosus tendon (SemiT double)
(4) single‐strand gracilis tendon (Gracilis single)
(5) double‐strand gracilis tendon (Gracilis double)
(6) single‐strand iliotibial band (ITB single)
(7) double‐strand iliotibial band (ITB double)
(8) superficial layer of the quadriceps tendon (Quad).

**Figure 2:**
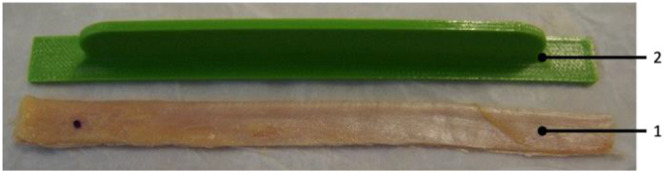
Photograph of a double‐strand iliotibial band graft (1) with a custom‐made 3D printed template (2) of a flat superficial medial collateral ligament configuration.

The specimens were stored at −20°C and thawed for 24 h at room temperature before anatomic dissection. Throughout preparation and testing, the specimens were kept moist with phosphate‐buffered saline to prevent tissue dehydration. The skin and subcutaneous tissue were removed, while leaving the fascia and muscles intact. Subsequently, the SemiT, Gracilis and Quad tendons, the ITB and the sMCL were harvested. The hamstring tendons were detached from their tibial insertion and bluntly separated from their muscle using a tendon stripper. Then, the sMCL was identified by anatomical dissection and excised as previously described [22, 23]. For this purpose, the sMCL was dissected from its tibial attachment up to its femoral attachment, being separated from the fibres of the posterior oblique ligament (POL) and deep medial collateral ligament (dMCL). As each layer and portion of the Quad tendon provides comparable biomechanical properties [5, 12, 21], only the central portion of the superficial layer of the Quad tendon was harvested using a protocol described by Chivot et al. [5]. Dissection and harvesting of the ITB were performed as described by previous studies [7, 11]. After identification of the ITB, its midportion was excised through a lateral longitudinal incision 13 mm proximal to the lateral femoral condyle and by sharp separation from the biceps femoris tendon [7, 11]. Both Quad and ITB were already harvested with the help of a 3D‐printed template (length 130.0 mm, width 11.8 mm) comparable to the size of the matched sMCL (Figure 2) and according to previous anatomical studies on the MCL [18], whereas the depth of the graft was predetermined by the individual specimen.

### Graft preparation and configuration

Once the tendon harvest was completed, the round hamstrings tendons were transformed into flat tendons according to previous technical descriptions of flat sMCL procedures [1, 9]. By doing so, the hamstring tendons were partially incised longitudinally and flattened using a raspatory to produce flat grafts [9]. Based on group randomization, the grafts were then either doubled (double‐strand groups) or retained as single‐strand grafts. Subsequently, the flattened grafts were shaped to a graft size comparable to the size of the matched sMCL using a custom‐made 3D‐printed template (length 130.0 mm, width 11.8 mm) (Figure 2) as mentioned above.

**Figure 3:**
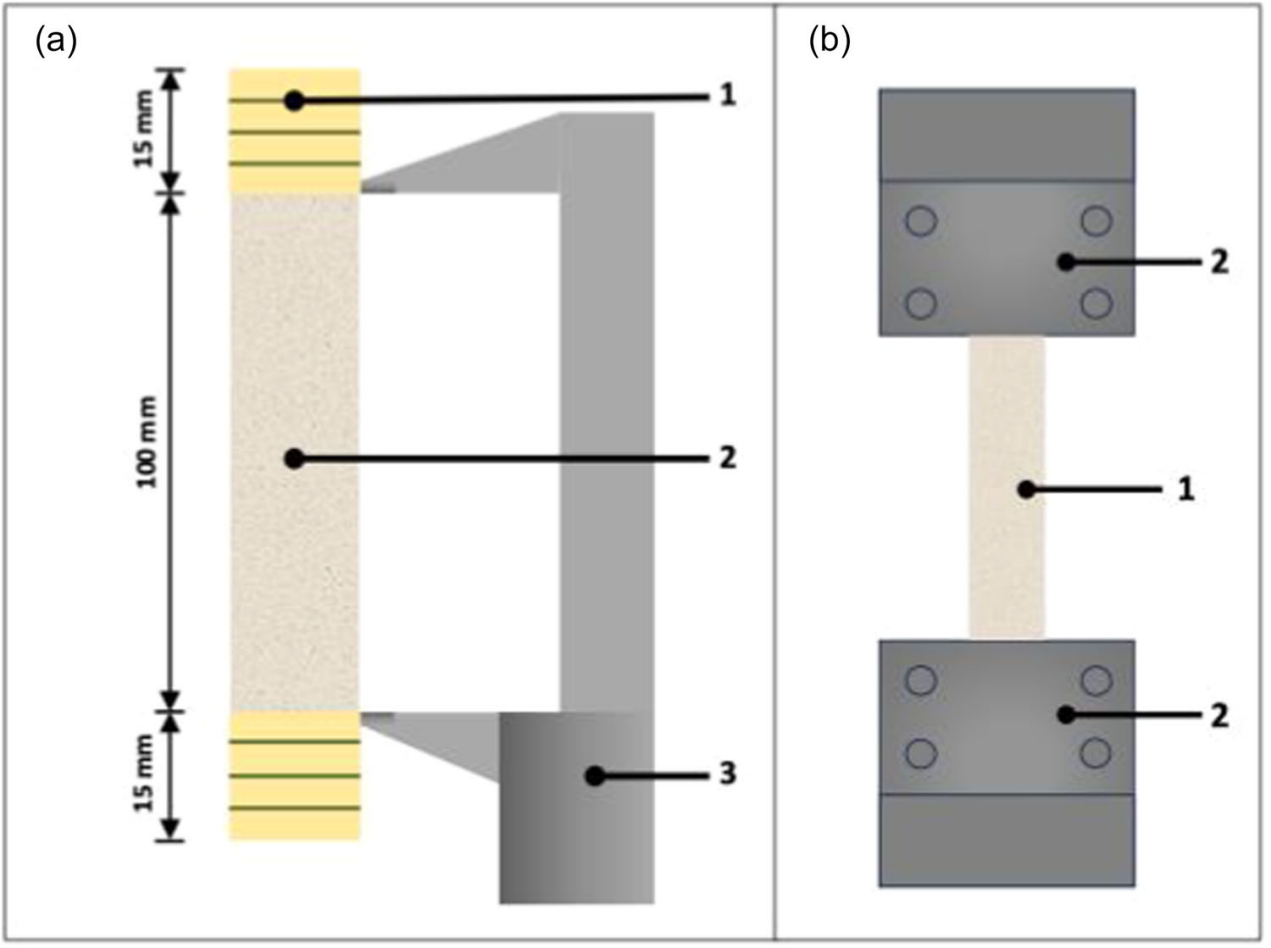
(a) Schematic illustration of a tendon graft with a 100 mm free tendon part (2) and 15 mm free ends reinforced with Krackow locking stitches (1) (braided coated Vicryl suture, Nr. 2, Ethicon); calliper for measurements (3). (b) Schematic illustration of the graft fixation in the uniaxial testing machine. (1) graft; (2) custom‐made mechanical clamps for fixation of the different grafts. Graft size in between the clamps is 100 mm (length) and 11.8 mm (width).

In all grafts, each 15‐mm free end of the grafts was reinforced with Krackow locking stitches (braided coated Vicryl suture Nr. 2, Ethicon) to provide accurate grip in the custom‐made clamps and thus prevent soft tissue slippage during biomechanical testing. In the double‐strand grafts, the graft was layered and at each 15 mm free end sutured together, whereas no further augmentation was performed mid‐substance. After preparation, a standardized and comparable graft size of 100.0 × 11.8 mm was achieved between the clamps for all groups, which was adopted from a previous study on the anatomy of the MCL [18] (Figure 3a). Finally, each reinforced, 15‐mm free end of the grafts was attached to a custom‐made clamp for biomechanical testing (Figure 3b).

### Biomechanical testing and data acquisition

**Figure 4:**
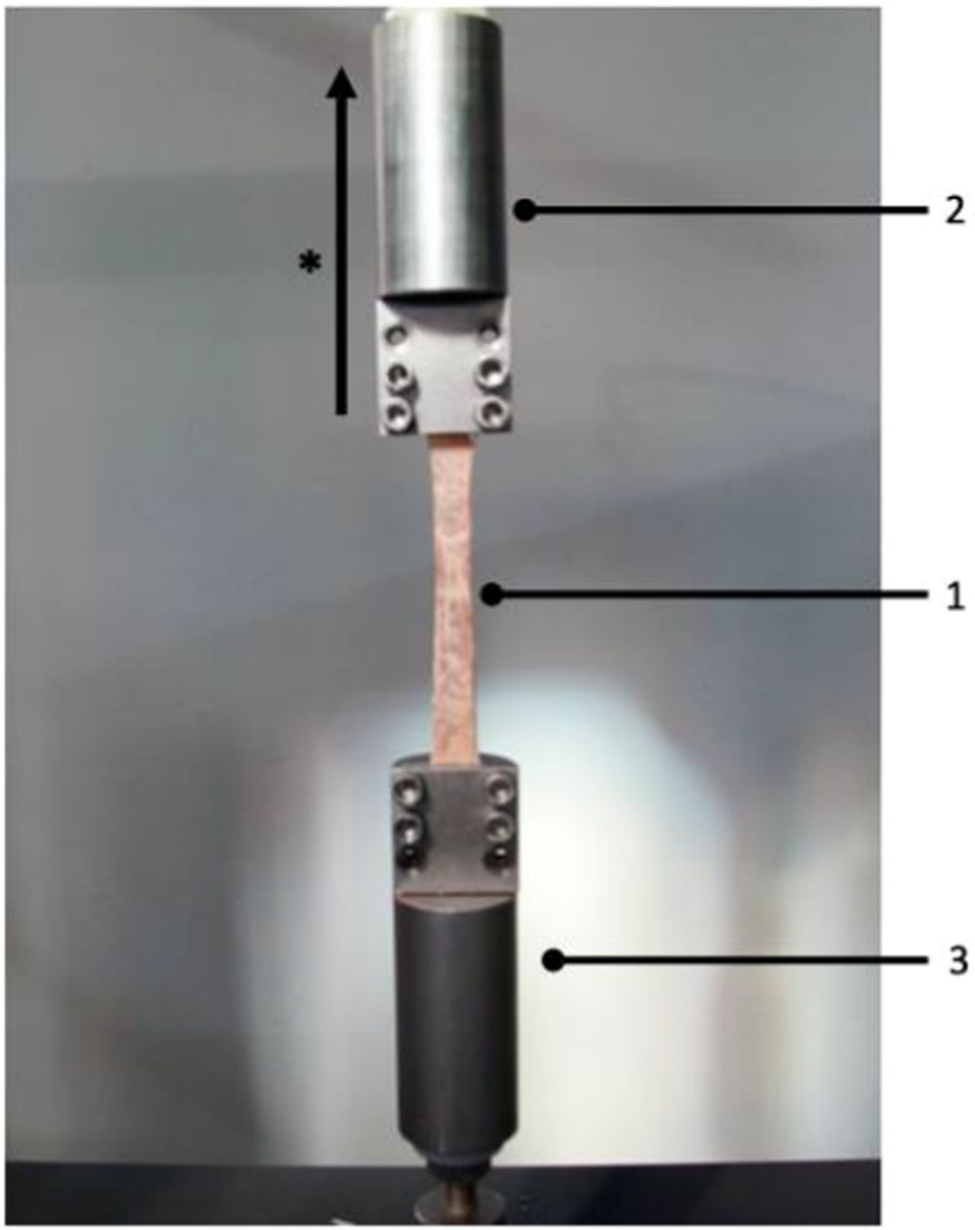
Photograph of the test set‐up in the universal uniaxial materials testing machine. A double‐strand iliotibial band graft (1) is positioned between the two clamps (2/3). Whereas clamp Nr. 3 is fixed to the base of the testing machine, uniaxial force is applied to the graft (1) via clamp Nr. 2 with the direction of the force according to the displayed arrow (*).

Biomechanical testing was performed using a uniaxial testing machine (Zwick/Roell Z005). The testing machine is calibrated with a distance resolution of 0.12 μm and repeatability of ±3.5 μm. One clamp was fixed to the base plate of the testing machine, while the second clamp was attached to the test actuator. The orientation of the tendon graft, and therefore the force vector, was perpendicular to the base plate of the testing machine, corresponding to a worst‐case loading scenario for an MCL graft (Figure 4). The testing protocol was adapted from a previous study evaluating the biomechanical properties of the primary medial knee ligaments [31]. Preconditioning of the grafts was performed with 10 cycles of tensile loading between 10 and 50 N at a rate of 20 mm/min, followed by subsequent loading to failure at 20 mm/min. Based on the actuator displacement and tensile loading forces, force–displacement curves were generated to calculate the biomechanical test parameters of each graft. The calculation of the individual parameters was carried out with the help of a custom‐made MATLAB script (Version R2024a, MathWorks) and defined as follows:

**Stiffness (N/mm)** was defined as the maximum slope of the linear loading range of the force–displacement curve during the load to failure test.

**Load to failure (N)** was defined as the maximum tensile load at which no further change in tendon length resulted in an increase or even a collapse in the load. The mode of failure was then defined when the peak drop in the load elongation curve occurred.

**Cross‐sectional area (mm** ^**2**^ **)** was measured by an electronic digital calliper (accuracy 0.01 mm) as depth (mm) × width (mm) at the midportion of each graft.

**The tensile stress (N/mm** ^**2**^ **)** was defined as the force per area at which the force is perpendicular to the beginning of the test.

**The elastic modulus (MPa)** was defined as the maximum gradient between two measuring points, which have three measuring points between them, during the tear‐out test in the stress‐strain curve.

**Ultimate Strain (%)** was defined as the elongation measured by the testing machine divided by the initial length.

**The Yield load (N)** was defined as the intersection point between the stress‐strain curve and the line of the modulus of elasticity, which is shifted by 0.1% elongation.

### Statistical analysis

An a priori power analysis was performed using G*Power‐2 software (University of Düsseldorf) [10]. Based on mean values and standard deviations from prior studies comparing the stiffness of different human cadaveric graft types (228 ± 103 and 280 ± 63.9 N/mm) [29], it was assumed that a sample size of *n* = 10 for each group would allow detection of changes of the stiffness (effect size/Cohen *d* = 1.4) with 80% power at the significance level of *p* < 0.05.

Statistical analysis was performed using GraphPad Prism 10 (Version 10.0.0). Descriptive data are presented as mean value ± standard deviation (SD). Normality of data distribution within each graft type was tested and proved using the Shapiro–Wilk test. A one‐way analysis of variance (ANOVA) with post hoc Tukey's multiple comparisons test was conducted to identify significant differences among the groups regarding stiffness, load to failure, cross‐sectional area, tensile stress, elastic modulus, ultimate strain and yield load. Overall level of significance was set at *p* < 0.05.

## RESULTS

### Effect of graft configuration

**Figure 5:**
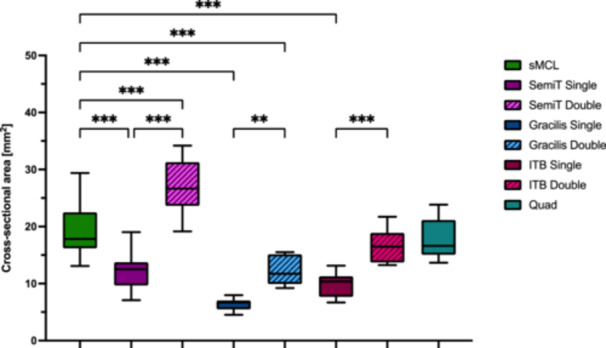
Cross‐sectional area (mm^2^) of the superficial medial collateral ligament (sMCL) and the different flat grafts. Significance is indicated by asterisks (**p ≤ 0.01; ***p ≤ 0.001). Gracilis double, double‐strand gracilis tendon; Gracilis single, single‐strand gracilis tendon; ITB double, double‐strand iliotibial band; ITB single, single‐strand iliotibial band; n.s., not significant; Quad, superficial layer of the quadriceps tendon; SemiT double, double‐strand semitendinosus tendon; SemiT single, single‐strand semitendinosus tendon.

**Figure 6:**
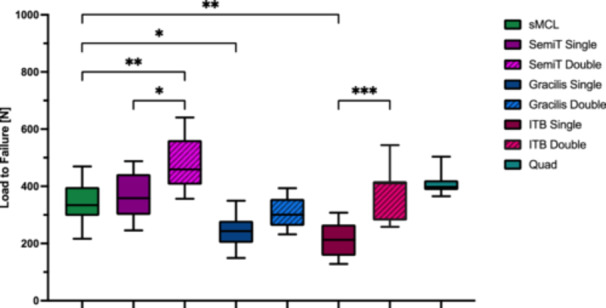
Load to failure (N) of the superficial medial collateral ligament (sMCL) and the different flat grafts. Significant differences are indicated by asterisks (*p ≤ 0.05; **p ≤ 0.01; ***p ≤ 0.001). Gracilis double, double‐strand gracilis tendon; Gracilis single, single‐strand gracilis tendon; ITB double, double‐strand iliotibial band; ITB single, single‐strand iliotibial band; n.s., not significant; Quad, superficial layer of the quadriceps tendon; SemiT double, double‐strand semitendinosus tendon; SemiT single, single‐strand semitendinosus tendon.

**Figure 7:**
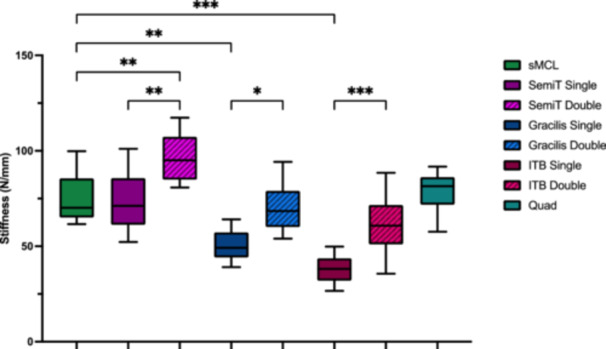
Stiffness (N/mm) of the superficial medial collateral ligament (sMCL) and the different flat grafts. Significance is indicated by asterisks (*p ≤ 0.05; **p ≤ 0.01; ***p ≤ 0.001). Gracilis double, double‐strand gracilis tendon; Gracilis single, single‐strand gracilis tendon; ITB double, double‐strand iliotibial band; ITB single, single‐strand iliotibial band; n.s., not significant; Quad, superficial layer of the quadriceps tendon; SemiT double, double‐strand semitendinosus tendon; SemiT single, single‐strand semitendinosus tendon.

**Figure 8:**
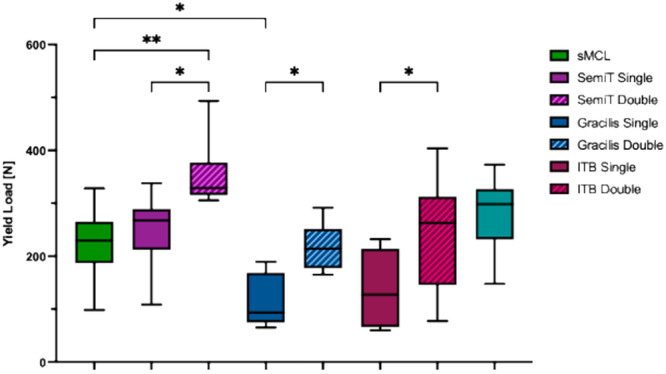
Yield load (N) of the superficial medial collateral ligament (sMCL) and the different flat grafts. Significance was indicated by asterisks (*p ≤ 0.05; **p ≤ 0.01). Gracilis double, double‐strand gracilis tendon; Gracilis single, single‐strand gracilis tendon; ITB double, double‐strand iliotibial band; ITB single, single‐strand iliotibial band; n.s., not significant; Quad, superficial layer of the quadriceps tendon; SemiT double, double‐strand semitendinosus tendon; SemiT single, single‐strand semitendinosus tendon.

Single‐strand grafts did not reproduce the cross‐sectional area of the native sMCL (*p* < 0.001), except for the quad tendon graft (not significant [n.s.]). Double‐strand grafts had a significantly higher cross‐sectional area compared to single‐strand grafts (*p* < 0.01). Whereas only the double‐strand ITB graft showed a comparable cross‐sectional area to the native sMCL (n.s.) (Table 1 and Figure 5). Graft configuration significantly affects the biomechanical properties of the flat grafts, as double‐strand configuration resulted in a significant increase in stiffness, load to failure, elastic modulus and yield load (*p* < 0.05) and a significant decrease in tensile stress and ultimate strain (*p* < 0.05) compared to single‐strand grafts (Table 1 and Figures 5, 6, 7, 8).

**Table 1:** Mean value ± standard deviation of the biomechanical parameters characterizing the different flat grafts, together with the *p*‐values in brackets following the statistical comparison of native superficial medial collateral ligament (sMCL) and the tested grafts.

	Stiffness (N/mm)	Load to failure (N)	Elastic modulus (MPa)	Yield load (N)	Tensile stress (N/mm^2^)	Ultimate strain (%)	Cross‐sectional area (mm^2^)
Native sMCL	74.4 ± 12.8	341.4 ± 73.2	2.6 ± 0.6	226.4 ± 66.5	1.8 ± 0.2	2.6 ± 0.7	19.3 ± 5.1
SemiT single	73.7 ± 15.0 (n.s.)	365.4 ± 76.9 (n.s.)	2.4 ± 0.5 (n.s.)	247.4 ± 68.4 (n.s.)	1.8 ± 0.2 (n.s.)	3.6 ± 1.0 (n.s.)	12.2 ± 3.3 (***p*** < **0.001**)
SemiT double	96.6 ± 13.2 (***p*** < **0.01**)	476.9 ± 89.6 (***p*** < **0.01**)	3.5 ± 0.9 (n.s.)	355.3 ± 58.9 (***p*** < **0.01**)	1.5 ± 0.1 (n.s.)	2.3 ± 0.6 (n.s.)	27.1 ± 4.7 (***p*** < **0.001**)
Gracilis single	50.9 ± 8.1 (***p*** < **0.01**)	242.6 ± 56.1 (***p*** < **0.05**)	0.8 ± 0.2 (***p*** < **0.001**)	115.6 ± 48.8 (***p*** < **0.05**)	1.5 ± 0.1 (***p*** < **0.05**)	4.5 ± 0.8 (***p*** < **0.001**)	6.3 ± 1.0 (***p*** < **0.001**)
Gracilis double	70.2 ± 12.8 (n.s.)	308.0 ± 56.2 (n.s.)	1.7 ± 0.5 (***p*** < **0.05**)	217.7 ± 42.9 (n.s.)	1.2 ± 0.2 (***p*** < **0.001**)	3.1 ± 0.7 (n.s.)	12.2 ± 2.4 (***p*** < **0.001**)
ITB single	37.8 ± 7.7 (***p*** < **0.001**)	212.4 ± 61.8 (***p*** > **0.01**)	1.3 ± 0.7 (***p*** < **0.001**)	132.3 ± 68.9 (n.s.)	1.6 ± 0.2 (n.s.)	2.8 ± 1.0 (n.s.)	9.8 ± 2.1 (***p*** < **0.001**)
ITB double	61.8 ± 15.6 (n.s.)	379.0 ± 89.4 (n.s.)	2.3 ± 0.8 (n.s.)	237.8 ± 101.0 (n.s.)	1.1 ± 0.2 (***p*** < **0.001**)	2.8 ± 0.6 (n.s.)	16.8 ± 2.9 (n.s.)
Quad	79.1 ± 9.9 (n.s.)	408.6 ± 39.2 (n.s.)	2.2 ± 0.5 (n.s.)	284.8 ± 66.8 (n.s.)	1.6 ± 0.7 (n.s.)	2.7 ± 0.6 (n.s.)	17.6 ± 3.5 (n.s.)

Abbreviations: Gracilis double, double‐strand gracilis tendon; Gracilis single, single‐strand gracilis tendon; ITB double, double‐strand iliotibial band; ITB single, single‐strand iliotibial band; n.s., not significant; Quad, superficial layer of the quadriceps tendon; SemiT double, double‐strand semitendinosus tendon; SemiT single, single‐strand semitendinosus tendon.

### Comparison with native sMCL

Only SemiT single and quad tendon grafts mirrored the biomechanical properties of the native sMCL, with comparable stiffness, load to failure, elastic modulus, yield load, tensile stress and ultimate strain (n.s.). In contrast, Gracilis double and ITB grafts demonstrated significantly lower tensile stress (*p* < 0.001) and elastic modulus (*p* < 0.05) than the native sMCL, while stiffness, load to failure, yield load and ultimate strain were comparable (n.s.). Doubling the SemiT increased its biomechanical properties, exhibiting significantly greater stiffness, load to failure and yield load compared to the native sMCL (*p* < 0.01). Conversely, single‐strand Gracilis and ITB grafts yielded significantly decreased biomechanical properties compared to the native sMCL, with significantly lower stiffness, load to failure, elastic modulus and yield load (*p* < 0.01) and higher tensile stress and ultimate strain (*p* < 0.001) (Table 1 and Figures 5, 6, 7, 8).

### Failure modes

As the peak drop in the loading curve was defined as failure of the graft, in all groups only mid‐substance tears were observed.

## DISCUSSION

The main finding of this study was that flattened single‐strand SemiT, double‐strand Gracilis, double‐strand ITB and partial‐thickness Quad tendon grafts best reproduced the biomechanical properties of the native sMCL and can therefore be considered as appropriate options for flat medial reconstructions from a biomechanical point of view. In contrast, single‐strand Gracilis and single‐strand ITB grafts demonstrated inferior biomechanical properties compared to the native sMCL, whereas doubled SemiT grafts surpassed the biomechanical properties of the native sMCL. Accordingly, the use of these grafts may result in an increased risk of residual medial knee laxity or medial overconstraint.

Previous biomechanical studies have analyzed the biomechanical properties of common soft tissue ACL grafts [14, 29]. As these grafts showed increased cross‐sectional areas between 49.1 and 81.4 mm^2^ [14], they are not reproducing the wide and broad cross‐sectional area of the native sMCL. In fact, anatomical studies of the native sMCL have shown significantly smaller cross‐sections of 24.5 mm^2^ [6], leading to the idea of anatomic MCL reconstructions with flat tendon grafts to best restore knee kinematics. Furthermore, biomechanical studies have demonstrated promising results with flat sMCL reconstructions, restoring native knee kinematics [8]. This study demonstrated that flattened human hamstring tendons do not directly replicate the cross‐sectional area of the sMCL [6] in either the single‐ or double‐strand technique (6.3–27.1 mm^2^), whereas doubled ITB and Quad tendon grafts might reproduce its flat anatomy with comparable cross‐sectional areas of 16.8 and 17.6 mm^2^, respectively. However, grafts should also replicate the biomechanical properties of the native ligament as the cross‐sectional area only describes morphology. Since stiffness reflects a material's resistance to deformation [35] and is a key determinant of its biomechanical properties, not all grafts demonstrated properties comparable to those of the native sMCL. In the present study, the native sMCL reached a stiffness comparable to previous investigations with 63.0–80.0 N/mm [6, 25, 31, 34]. This was only achieved by SemiT‐single, Quad, Gracilis‐double and ITB‐double grafts, whereas Gracilis‐single and ITB‐single grafts only reached 37.8 and 50.9 N/mm, respectively. This could lead to graft laxity and secondary insufficiency during early rehabilitation, according to the forces described by Shelburne et al. [26], which could determine to go stiffer in the primary setting with the viable graft options. On the other hand, grafts exceeding the stiffness of the native sMCL, such as the SemiT‐double (96.6 N/mm), might increase the risk of over constraint, whereas this might be neglected if the graft is positioned correctly [17, 33]. This illustrates the effect of double versus single‐strand configurations in flattened hamstring and ITB grafts, as doubling the graft results in a significant increase in stiffness. Nevertheless, it has to be mentioned that in cases of medial over constraint due to increased graft stiffness, this might be negligible due to secondary graft loosening, according to Shelburne et al. [26].

Regarding yield‐load, in our study, the sMCL reached 226.4 ± 66.5 N, which was nearly mimicked by Quad, SemiT‐single, ITB‐double and Gracilis‐double, ranging from 217.7 ± 42.9 to 284.8 ± 66.8 N whereas underscored by Gracilis‐ and ITB‐single and exceled by SemiT‐double. Concerning the elastic modulus, our results do not match the results presented by Cho et al. [6] with 326.75 ± 225.22 MPa for the native MCL, and in our study, we presented 2.6 ± 0.6 MPa regarding the native sMCL. But, in their study, they performed a totally different testing setup as they tested the originally attached complete MCL tibial and femoral, whereas in our study, we tested the isolated sMCL and tested it detached. Nevertheless, also regarding the elastic modulus, Quad, SemiT‐single, ITB‐double and Gracilis‐double mimic the elastic modulus of the sMCL, whereas SemiT‐single excel and ITB and Gracilis‐single underscore the native sMCL. Concerning LTF, previous studies revealed a maximum LTF of the native sMCL between 465.0 and 799.0 N [6, 20, 25, 31, 34], whereas our results remained inferior with 341.4 N. Given this, we may be underestimating the biomechanical properties of the native sMCL in the present study, but different configurations and different biomechanical test setups should be considered when interpreting the results. As our study is, to our knowledge, the first cadaveric study to analyze flattened SemiT and Gracilis, previous studies have already investigated ITB double and partial thickness quad grafts at 1160 and 972 N in LTF [29]. As mentioned above, these are higher than our results and even higher than the native sMCL in previous studies [6, 25, 31, 34]. This highlights the need for the native sMCL as a control group when comparing grafts that best mimic its native structure. Similar findings regarding SemiT‐single and Quad best mimicking the native sMCL, followed by Gracilis‐double and ITB‐double, were observed for all other biomechanical properties analyzed in this study.

As the flat sMCL technique showing promising biomechanical results [8], our study now provides biomechanical properties of possible graft choices for flat sMCL repair. The results of the present study are of high clinical relevance, as one key finding shows, that the properties of the graft can be adapted to the biomechanical requirements for flat sMCL reconstruction by the type of preparation. Gracilis‐single and ITB‐single do not seem to be adequate graft options from a biomechanical point of view as there might be a risk of residual instability. Whereas, on the other hand, the doubling of these grafts improves their biomechanical properties and cross‐sectional area converging to mimicking the native sMCL [6, 20, 25, 31, 34]. However, doubling the SemiT leads to exceeded properties, so that there is concern about medial over constraint [27, 28]. In conclusion, SemiT‐single and Quad appear to be recommended based on the best possible imitation of the native sMCL biomechanical behaviour, but further studies are needed, particularly biomechanical studies with flat sMCL reconstructions comparing those graft options.

This study had several limitations inherent to in‐vitro biomechanical studies that must be considered before transferring our results into clinical practice. The age of the used specimens is higher compared to most patients seen in the clinical setting, who suffer from these injuries and need surgical repair. One important limitation certainly is that only linear loads were analyzed with this setup, and that no cyclic loading was performed for analyzing biomechanical properties such as cyclic displacement or tissue hysteresis prior to LTF testing. Therefore, a certain grade of tissue hysteresis during repetitive loading cannot be analyzed with our setup. Concerning the grafts, different donor sites of the ITB itself might influence its primary biomechanical properties, which were not tested in this study. Regarding measurements, the cross‐sectional area was only performed with a digital calliper, as 3D scanning was not available, which might have affected the precision of measurements. Furthermore, healing of the grafts was not considered as in vivo even in a short time frame, the results could differ due to healing and scarring of the structures. Additionally, different grafts might make different fixation methods necessary, which clearly differ in their biomechanical properties as well and the fact that no control group for the flattened hamstring tendons was performed. In addition, it must be mentioned, that further graft options for flat sMCL reconstructions such as the peroneus longus, tibialis anterior and Achilles tendon, were not able to be tested due to limited graft availability. Furthermore, it must be mentioned that parameters were calculated indirectly via the crossbar of the uniaxial material testing machine and the loading cell rather than using optical measurement techniques mid‐substance, therefore optical strain was not measured, which might have influenced the outcome. Especially as the graft length between the clamps was 100 mm. Yet, the study set‐up was in line with previous studies analyzing biomechanical properties of specific grafts providing comparable conditions [5, 9, 29].

## CONCLUSION

The graft type and configuration of the grafts significantly affects the biomechanical properties of flat sMCL grafts. Single‐strand SemiT and Quad, and double‐strand Gracilis and ITB grafts, mimic the biomechanical properties of the native sMCL, representing viable options for flat sMCL reconstructions. In contrast, double‐strand SemiT grafts exceeded these properties, while single‐strand Gracilis and ITB grafts demonstrate decreased biomechanical properties, possibly resulting in increased risk of medial overconstraint or residual laxity.

## AUTHOR CONTRIBUTIONS

Conception and design, testing and data acquisition, statistical analysis and writing: Thorben Briese, Christian Peez and Elmar Herbst. Testing and data acquisition, statistical analysis: Philipp Runde. Internal review and data acquisition: Matthias Klimek. Internal review: Adrian Deichsel and Michael J. Raschke. Internal review, conception and design: Christoph Kittl.

## CONFLICT OF INTEREST STATEMENT

Elmar Herbst is Deputy Editor‐in‐Chief for the *Knee Surgery, Sports Traumatology and Arthroscopy* (KSSTA). Adrian Deichsel is Web Editor for the KSSTA. The remaining authors declare no conflicts of interest.

## ETHICS STATEMENT

The specimens were dissected and biomechanically tested under the approval of the Institutional Ethics Committee of the University of Muenster (IRB reference number 2020‐181‐f‐S).

## Data Availability

Data are available from the corresponding author upon reasonable request.
